# Headache comorbidity in epilepsy and functional/dissociative seizures: an exploratory cross-sectional study in a tertiary epilepsy center

**DOI:** 10.3389/fneur.2026.1797757

**Published:** 2026-03-16

**Authors:** Marvin Jüchtern, Katharina Timpte, Guido Widman, Yvonne Weber, Stefan Wolking

**Affiliations:** 1 University Clinic of Neurology, Evangelical Hospital, Carl von Ossietzky Universität Oldenburg, Oldenburg, Germany; 2 Division of Clinical Cognitive Sciences, Department of Neurology, RWTH Aachen University Hospital, Aachen, Germany; 3 Section of Epileptology, Department of Neurology, RWTH Aachen University Hospital, Aachen, Germany

**Keywords:** comorbidity, fds, medication, migraine, paroxysmal disease, seizure

## Abstract

**Introduction:**

Headache disorders are common in patients with epilepsy and functional/dissociative seizures (FDS) and may influence quality of life and clinical outcomes. Migraine has been proposed as a reciprocal risk factor for epileptic and functional seizures. We aimed to determine the prevalence of headache disorders and associated clinical factors in a cohort of patients undergoing video-EEG monitoring at a tertiary epilepsy center.

**Methods:**

In this retrospective cross-sectional study, clinical data from 164 adult inpatients evaluated in an epilepsy monitoring unit were analyzed. Headache diagnoses were established using a structured interview based on International Classification of Headache Disorders (ICHD-3) criteria. Associations between headache disorders and clinical variables – including epilepsy subtype, seizure localization, antiseizure medication (ASM), and pre-existing illnesses – were assessed using Fisher’s exact tests and logistic regression analyses.

**Results:**

Seventy percent of patients were diagnosed with epilepsy and 13% with FDS. Among epilepsy patients, 48% reported headache (21% migraine, 18% tension-type headache), whereas 77% of patients with FDS reported headache (50% migraine, 14% tension-type headache). Epilepsy overall, particularly lesional focal epilepsy, was negatively associated with headache and migraine compared with no-epilepsy patients. In contrast, FDS was positively associated with headache and migraine. Migraine with aura was not disproportionately represented in epilepsy or FDS. Female sex and younger age were associated with headache. Levetiracetam and valproic acid use were negatively associated with migraine. Frontal seizure onset was negatively associated with migraine, whereas temporal onset showed a non-significant positive trend. Obesity was positively associated with migraine with aura, while depression showed a negative association with migraine.

**Discussion:**

Headache disorders are frequent in patients undergoing evaluation for paroxysmal neurological disorders, particularly in FDS. Systematic headache screening—especially in young and female patients—may help reduce disease burden. Further studies should clarify shared mechanisms and potential therapeutic implications.

## 1 Introduction

Epilepsy ranks among the most common neurological conditions worldwide, affecting approximately 1% of the global population (1). Comorbidities are highly prevalent in people with epilepsy, and conditions such as psychiatric disorders, rheumatic and cardiovascular disease, and pain syndromes occur more frequently than in the general population (2). Comorbidities, including headache disorders, are associated with poorer prognosis and reduced quality of life (3).

According to the Global Burden of Disease study, approximately 1–2 per 1,000 individuals are affected by both epilepsy and migraine (4). An association between epilepsy and headache disorders has long been suggested. A meta-analysis reported that people with epilepsy have a more than a 50% increased lifetime risk of migraine (5). Previous studies have shown that approximately one quarter of people with epilepsy experience recurrent headache symptoms, and 15% exhibit migraine features (6). In a larger study, 41% of patients with idiopathic generalized epilepsy reported migraine (7). Tension-type headache (TTH) has been reported in between 15 to 33% of people with epilepsy (3, 7–9). However, other investigations, including multicenter studies, have not found significant associations between epilepsy and headache disorders (10–12). A systematic review and meta-analysis published in 2015 noted that only a limited number of studies applied rigorous diagnostic criteria or adequately controlled for confounding factors. When focusing on higher quality studies, the increased prevalence of migraine in people with epilepsy, and vice versa, appears to be robust (5).

Several shared pathophysiological mechanisms may link epilepsy and headache disorders, including cortical spreading depression (CSD), altered neurotransmitter release (particularly glutamate and gamma-aminobutyric acid), common genetic factors, similar triggering factors (e.g., sleep deprivation and hormonal and toxic influences), and overlapping pharmacological treatments (13). For example, variants in *CACNA1A*, *SCN1A*, and *ATP1A2* are associated with familial hemiplegic migraine and genetic epilepsy syndromes (14, 15). A key pathophysiological link may involve neuronal hyperexcitability and the resulting CSD due to dysfunctional ion channel activity in the central nervous system.

In people with functional/dissociative seizures (FDS), the most common differential diagnosis of epilepsy, an increased prevalence of headache and pain has also been reported (16). A recent meta-analysis found that nearly a third of individuals with FDS had migraine, representing almost a threefold higher prevalence compared with people with epilepsy (17). Another study reported longer migraine attacks and more frequent non-visual aura symptoms in individuals with FDS (18).

In this study, we analyzed demographic and clinical characteristics of patients undergoing comprehensive diagnostic evaluation in the epilepsy monitoring unit of a tertiary epilepsy center. The aim was to assess the prevalence of different headache disorders and to identify associated clinical factors.

## 2 Materials and methods

### 2.1 Data acquisition

All clinical data were collected from adult inpatients admitted to the epilepsy center at RWTH Aachen University Hospital over a one-year period. All patients underwent at least 3 days of continuous video-EEG monitoring in a dedicated epilepsy monitoring unit (EMU). Patients with moderate or severe intellectual disability, speech impairment due to neurological disorders, or insufficient language proficiency were excluded. Clinical data were obtained during the admission interview, from electronic medical records, and through a semi-structured interview conducted by physicians with clinical expertise in epileptology and headache disorders. Additional information was collected throughout the inpatient stay. All data were retrospectively reviewed and analyzed by the treating physicians.

This study was conducted in accordance with the ethical standards of the institutional and national research committees and the 1964 Helsinki Declaration and its later amendments. Ethical approval was obtained from the Ethics Committee of RWTH Aachen University (EK 24-046, CTC-A-Nr. 24-075).

### 2.2 Definition of medical terms

Patients with epilepsy were categorized into four groups: lesional focal epilepsy (LFE), defined by the presence of an epileptogenic lesion on magnetic resonance imaging (MRI); non-lesional focal epilepsy (NLFE), defined by the absence of an epileptogenic MRI lesion; generalized epilepsy (GE), including idiopathic generalized epilepsies; and other epilepsies (OE). The latter category included epilepsy syndromes such as developmental and epileptic encephalopathies.

Patients with acute symptomatic seizures (ASS) who did not meet the diagnostic criteria for epilepsy were classified in the no-epilepsy (NEP) group. This group also included patients with functional/dissociative seizures and other non-epileptic paroxysmal events.

Headache disorders were diagnosed according to the criteria of the current International Classification of Headache Disorders, 3rd edition (ICHD-3) (19). Headache categories included migraine, migraine with aura (MwA), migraine without aura (MwoA), tension-type headache (TTH) and other primary headache disorders (OH). The latter included, for example, trigeminal autonomic cephalalgias. Secondary headache disorders, cranial neuropathies, and facial pain syndromes listed in Parts Two and Three of the ICHD-3 were not included in the present analysis.

### 2.3 Statistical analysis

Associations between clinical variables and headache disorders were analyzed using Fisher’s exact test, with a significance level of 0.05. Cramér’s V (CV) was calculated to estimate effect size, using threshold values of ≥0.1, ≥0.3, and ≥0.5 to indicate weak, moderate, and strong associations, respectively. For 2 × 2 contingency tables, odds ratios (OR) with 95% confidence intervals (CI) were calculated to determine the direction and strength of associations.

For the entire cohort, associations between headache variables (any headache, migraine, migraine with aura [MwA], migraine without aura [MwoA], and tension-type headache [TTH]) and clinical variables were analyzed. Clinical variables included diagnosis, sex, antiseizure medication use (ASM; ever used), and pre-existing illnesses (PEI; ever diagnosed). Only the five most commonly used ASMs (lacosamide, lamotrigine, levetiracetam, topiramate, and valproic acid) and the five most common PEIs (cerebrovascular accident, depression, arterial hypertension, obesity, and traumatic brain injury) were included in the analysis.

For patients with epilepsy, additional analyses were performed to assess associations between headache variables and localization of seizure onset (LSO) as well as seizure types (ToS), classified according to the International League Against Epilepsy (ILAE) nomenclature (20). Generalized seizure types were not analyzed separately, as all such cases corresponded to a diagnosis of generalized epilepsy, which was already included as an independent variable in the full cohort analysis. LSO was determined based on EEG findings (focal slowing or epileptiform activity), MRI findings, or clearly localizing early semiological features. Due to small subgroup sizes, parietal and occipital LSO were excluded from the analysis.

Binomial logistic regression analyses were performed to evaluate the effect of age (independent variable) on the presence of headache and headache subtypes (dependent variables). Model fit was assessed using likelihood-ratio chi-square tests (*χ*^2^, significance level 0.05), and ORs with 95% confidence intervals were calculated. The proportion of variance explained by the model was estimated using Nagelkerke’s pseudo coefficient of determination (pseudo-*R*^2^), with values closer to 1 indicating stronger predictive performance.

No correction for multiple comparisons was applied, as this was an exploratory analysis involving largely independent variables and relatively small diagnostic subgroups.

All statistical analyses were performed using the open-source R-based software jamovi (The jamovi project (2025). Version 2.6.26)^1^.

## 3 Results

### 3.1 Whole-cohort data

A total of 164 patients were included in the study. Of these, 90 (55%) were female, and the mean age was 45.7 years (standard deviation [SD] 17.9). Headache disorders were present in 89 patients (54%). Migraine was the most prevalent headache disorder, affecting 45 patients (27%), followed by tension-type headache (TTH), which was reported by 27 patients (16%). Among patients with migraine, 16 (10%) had migraine with aura (MwA) and 29 (18%) had migraine without aura (MwoA).

Headache disorders were more common in female patients. Among those with migraine, 78% were female, including 79% of patients with MwoA. Similarly, 67% of patients with TTH and 67% of patients with any headache disorder were female. No patient met the ICHD-3 criteria for chronic migraine. Five patients (3%) were diagnosed with other primary headache disorders, including cluster headache and epicrania fugax (ICHD-3 A4.11). Three patients were diagnosed with secondary headache disorders. An additional 13 patients (8%) reported recurrent headache symptoms that did not fulfill ICHD-3 diagnostic criteria.

Among the 89 patients with headache disorders, 57% reported using acute headache medication, primarily nonsteroidal anti-inflammatory drugs. Preventive migraine treatment was used by 9% of patients. Eight patients (5%) reported a temporal association of their headaches with seizures, including one functional/dissociative seizure patient with prodromal (migrainous) headache. Three cases of ASM-associated headache were reported, concerning levetiracetam, lamotrigine and sultiame.

For more detailed information please refer to Table 1.

**Table 1:** Descriptive data of the whole cohort as well as epilepsy and no-epilepsy patients.

Clinical variables	Whole cohort	Epilepsy patients	No-epilepsy patients
	*n* = 164	*n* = 115	*n* = 49
Mean age	45.7 years	46.3 years	44.4 years
Female sex	90 (54.9%)	60 (52.2%)	30 (61.2%)
Diagnosis			
Focal epilepsy		92 (80.0%)	
Lesional focal epilepsy		51 (44.3%)	
Non-lesional focal epilepsy		41 (35.7%)	
Generalized epilepsy		16 (13.9%)	
Idiopathic generalized epilepsy		8 (7.0%)	
Other epilepsy		7 (6.1%)	
Functional/dissociative seizures			22 (44.9%)
Vertigo, dizziness or (Pre)Syncope			9 (18.4%)
Acute symptomatic seizure			13 (26.5%)^a^
Other non-epileptic diagnosis			6 (12.2%)
Headache			
Headache overall	89 (54.3%)	55 (47.8%)	34 (69.4%)
Migraine	45 (27.4%)	24 (20.9%)	21 (42.9%)
Migraine with aura	16 (9.8%)	6 (5.2%)	10 (20.4%)
Migraine without aura	29 (17.7%)	18 (15.7%)	11 (22.4%)
Tension-type headache	27 (16.5%)^b^	21 (18.3%)^b^	6 (12.2%)
Other headache	5 (3.0%)	2 (1.7%)	3 (6.1%)
Headache n.o.s.	13 (7.9%)	9 (7.8%)	4 (8.2%)
Diagnosis prior to admission^*^			
Migraine with aura	16 (100%)	6 (100%)	10 (100%)
Migraine without aura	12 (41.4%)	7 (38.9%)	5 (50.0%)
Tension-type headache	3 (11.1%)	1 (4.8%)	2 (33.3%)
Associated with seizure	8 (4.9%)	7 (6.1%)	1 (2.0%)
Associated with ASM	3 (1.8%)	2 (1.7%)	1 (2.0%)
Medication			
Antiseizure medication (ASM) overall	123 (75.0%)	99 (86.1%)	24 (49.0%)
Levetiracetam	90 (54.9%)	72 (62.6%)	18 (36.7%)
Lamotrigine	47 (28.7%)	38 (33.0%)	9 (18.4%)
Lacosamide	35 (21.3%)	32 (27.8%)	3 (6.1%)
Valproic acid	31 (18.9%)	29 (25.2%)	2 (4.1%)
Topiramate	22 (13.4%)	22 (19.1%)	0
>2 ASM	43 (26.2%)	41 (35.7%)	2 (4.1%)
Acute headache medication^#^	51 (57.3%)	32 (58.2%)	19 (55.9%)
Headache prophylaxis^#^	8 (9.0%)	4 (7.3%)	4 (11.8%)
Pre-existing illness			
High blood pressure	44 (26.8%)	30 (26.1%)	14 (28.6%)
Depression	32 (19.5%)	24 (20.9%)	8 (16.3%)
Obesity	7 (4.3%)	3 (2.6%)	4 (8.2%)
s/p Traumatic brain injury	11 (6.7%)	8 (7.0%)	3 (6.1%)
s/p Cerebrovascular accident	17 (10.4%)	13 (11.3%)	4 (8.2%)
Localization of seizure onset			
Frontal		25 (21.7%)	
Temporal		60 (52.2%)	
Parietal		4 (3.5%)	
Occipital		6 (5.2%)	
Type of seizure (ToS)			
Focal seizure			
With preserved consciousness		35 (30.4%)	
With impaired consciousness		59 (51.3%)	
To bilateral tonic clonic		58 (50.4%)	
Generalized seizure			
Tonic–clonic		12 (10.4%)	
Absence		9 (7.8%)	
Myoclonic		2 (1.7%)	
>2 different ToS		52 (45.2%)	

n.o.s., not otherwise specified; s/p, status post.

* Among those with the respective headache condition.

# Among those with a headache condition.

a One case who also presented with functional/dissociative seizures.

b One case who also presented with migraine (without aura).

### 3.2 Epilepsy patients

Patients with epilepsy were slightly older than the overall cohort, with a mean age of 46.3 years (SD 18.0), and 52% were female. Ninety-two patients (80%) were diagnosed with focal epilepsy, of whom 55% had lesional focal epilepsy. Sixteen patients (14%) were diagnosed with generalized epilepsy, half of whom were classified as having idiopathic generalized epilepsy. Three patients were diagnosed with other epilepsy syndromes, including Dravet syndrome, new-onset refractory status epilepticus/febrile infection-related epilepsy syndrome (NORSE/FIRES), and mitochondrial disease. Four patients presented with a first seizure at the time of admission without sufficient evidence for classification into a specific epilepsy subtype and were therefore categorized as having other epilepsy (OE).

The most commonly reported seizure types were focal impaired consciousness seizures (FIC), occurring in 51% of patients, followed by focal preserved consciousness seizures (FPC) in 30%, focal-to-bilateral tonic–clonic seizures (FBTC) in 50%, and generalized tonic–clonic seizures (GTC) in 10%. Other seizure types included absence seizures, myoclonic seizures, and status epilepticus. Overall, 45% of patients experienced more than one seizure type. The most frequent anatomical origin of seizures was temporal (38% of epilepsy cases), followed by frontotemporal (22%). In 34% of cases, the seizure onset zone could not be determined, including patients with generalized epilepsy.

Overall, 55 patients with epilepsy (48%) had a comorbid headache disorder. Migraine was present in 21% of patients (5% MwA, 16% MwoA), and TTH was present in 18%. Headaches temporally associated with ASM use or seizures were reported by 8% of patients.

Among patients with focal epilepsy, 47% had headache disorders, including 18% with migraine and 18% with TTH. Migraine was more common in patients with non-lesional focal epilepsy than in those with lesional focal epilepsy (24% vs. 14%), whereas TTH was less common (15% vs. 22%, respectively). Among patients with generalized epilepsy, 56% had headache disorders, including 38% with migraine and 19% with TTH.

### 3.3 No-epilepsy patients

The no-epilepsy (NEP) group included 22 patients with functional/dissociative seizures (FDS) (45%), 13 patients with acute symptomatic seizures following cerebrovascular events (27%), and 9 patients with syncope, most of whom presented with convulsive syncope (18%). The remaining patients were diagnosed with other neurological or psychiatric conditions, including paroxysmal dyskinesia. The mean age was 44.4 years (SD 17.5), and 61% of patients were female.

Notably, 49% of patients in the NEP group were receiving antiseizure medication (ASM) at the time of admission, largely driven by a treatment rate of 92% among patients with acute symptomatic seizures. Levetiracetam was the most commonly prescribed ASM in this group (37%).

**Figure 1:**
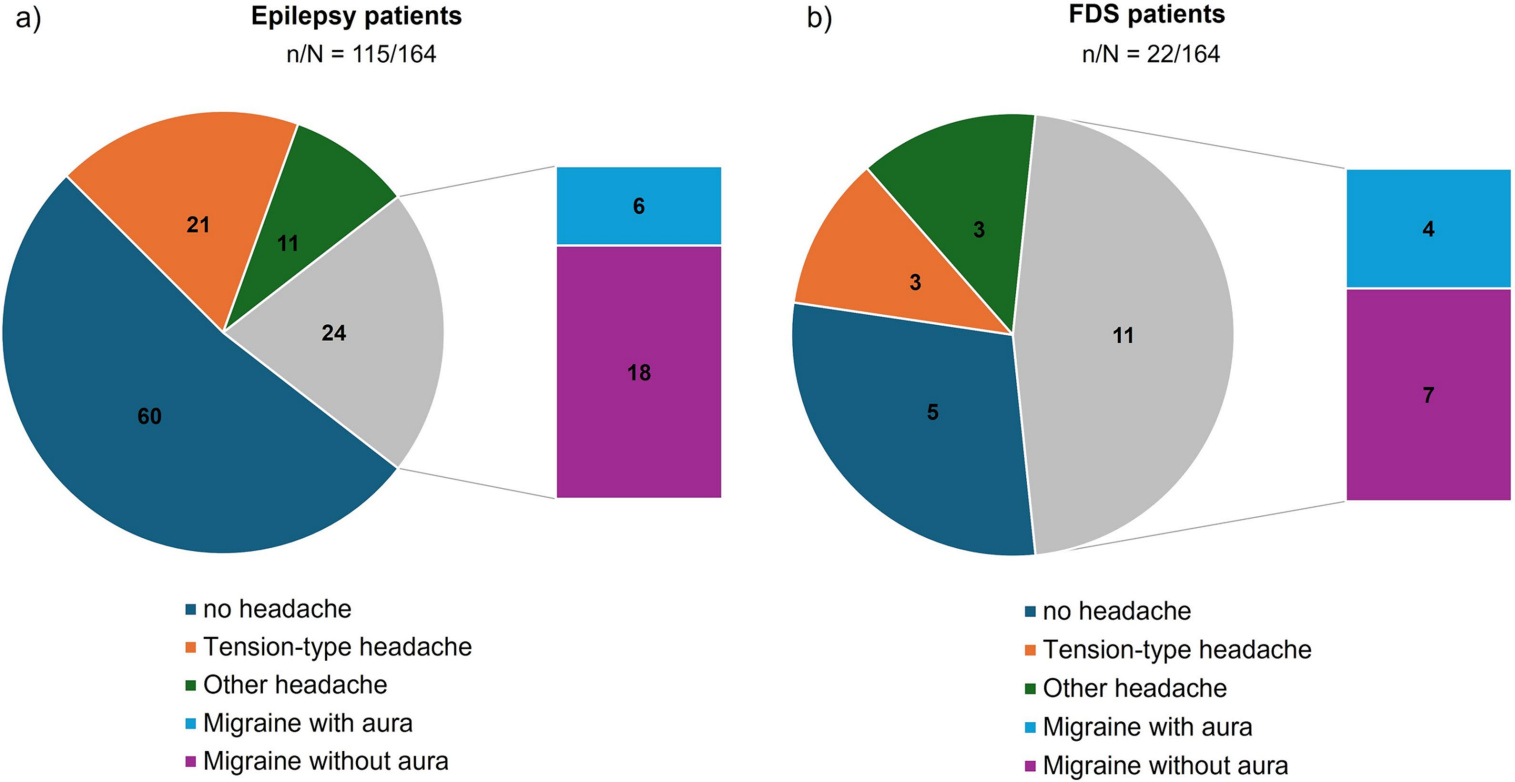
Types of headache disorders among epilepsy and FDS patients. Absolute shares of tension-type headache (orange), other headache (dark green) as well as migraine with aura (light blue) and migraine without aura (purple) among patients with epilepsy **(a)** and FDS **(b)**. The size of each chart segment represents the percentage of patients with the respective headache diagnosis among all patients of the subgroup. The numbers within each colored area give the absolute number of patients with the respective headache diagnosis in the subgroup. Note that migraine in total is given in gray.

Overall, 34 patients in the NEP group (69%) reported recurrent headache symptoms. Migraine was diagnosed in 43% of patients, including 20% with migraine with aura and 23% with migraine without aura, while 12% met diagnostic criteria for tension-type headache, as shown in Figure 1.

Among patients with FDS, 77% reported headache disorders, including 50% with migraine. Of those with migraine, 64% had migraine without aura. Tension-type headache was present in 14% of patients with FDS.

Figure 1 depicts the proportions of different headache disorders among epilepsy and FDS patients. For more detailed information please also refer to Table 1.

### 3.4 Association of epilepsy, functional/dissociative seizures and headache disorders

**Figure 2:**
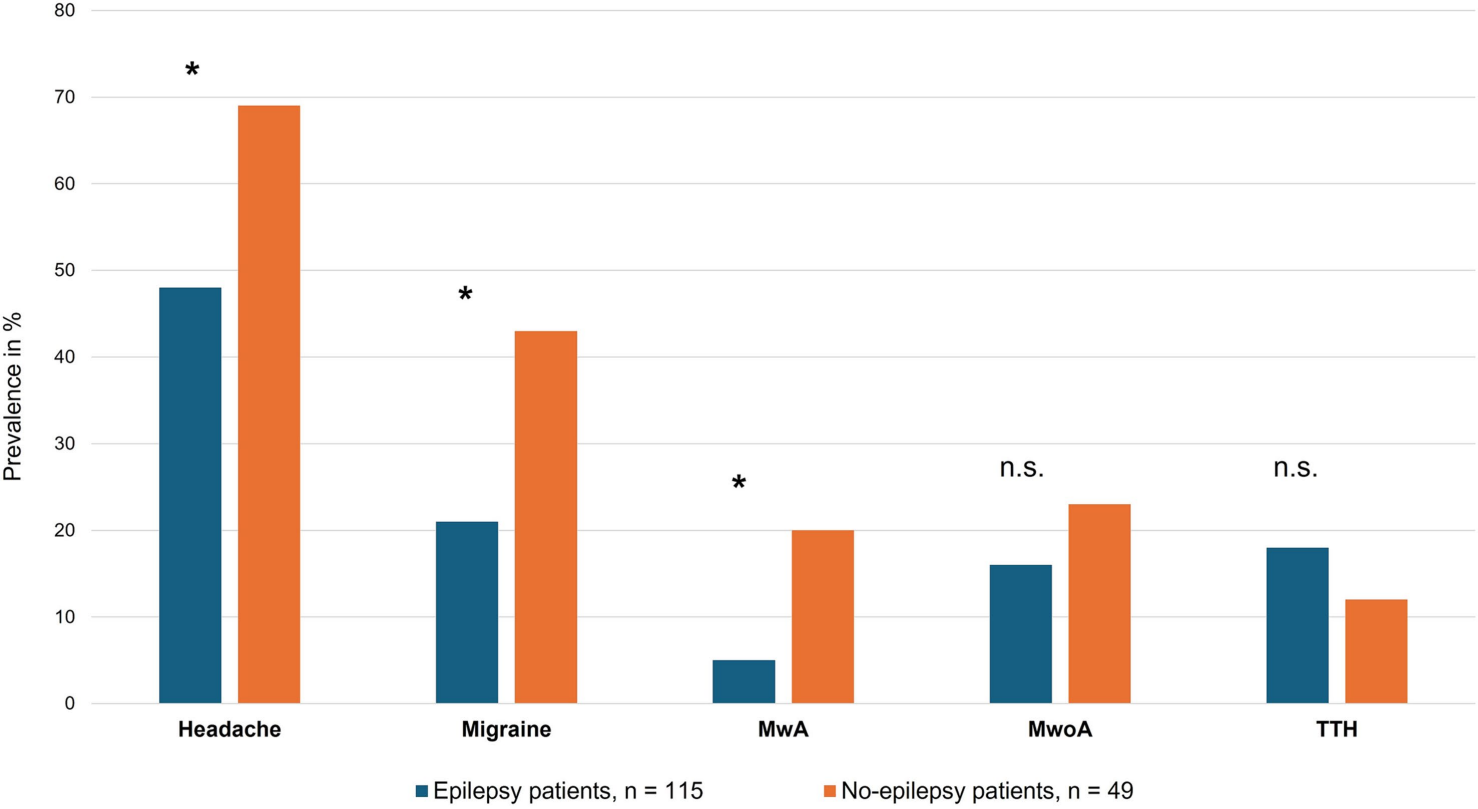
Comparison of headache prevalence in epilepsy and non-epilepsy patients. Differences in prevalence of headache in general as well as specific headache disorders among epilepsy (dark blue) and no-epilepsy (orange) patients. Prevalence in percentage of the respective group. *, significant; “n.s.”, not significant.

Binary Fisher’s exact tests revealed a significant negative association between epilepsy and the presence of any headache disorder (*p* = 0.016, OR 0.404, CV = 0.198), indicating that headache disorders were less common among patients with epilepsy than among those without epilepsy. Similarly, epilepsy was significantly negatively associated with migraine overall (*p* = 0.007, OR 0.352, CV = 0.226) and with migraine with aura (*p* = 0.007, OR 0.614, CV = 0.234). Negative associations were also observed between epilepsy and migraine without aura, whereas epilepsy and tension-type headache showed a positive association; however, these findings did not reach statistical significance. Detailed results are shown in Figure 2.

Within the epilepsy subgroups, focal epilepsy was significantly negatively associated with migraine (*p* = 0.005, OR 0.356, CV = 0.227) and migraine with aura (*p* = 0.002, OR 0.153, CV = 0.247). Lesional focal epilepsy showed similar negative associations with migraine (*p* = 0.008, OR 0.314, CV = 0.206) and migraine with aura (*p* = 0.003, OR 0.057, CV = 0.221). Non-lesional focal epilepsy showed non-significant trends toward negative associations with headache disorders. In contrast, generalized epilepsy showed positive, but non-significant, associations with headache disorders and small effect sizes, likely reflecting the limited number of cases.

Functional/dissociative seizures (FDS) were significantly positively associated with headache disorders overall (*p* = 0.022, OR 3.31, CV = 0.182) and with migraine (*p* = 0.019, OR 3.18, CV = 0.112) in the full cohort. In contrast, FDS showed a non-significant trend toward a negative association with tension-type headache (CV = 0.036). When compared specifically with non-FDS patients within the NEP group, FDS showed a non-significant trend toward a positive association with migraine without aura (*p* = 0.185, OR 2.68).

### 3.5 Variables associated with headache

**Figure 3:**
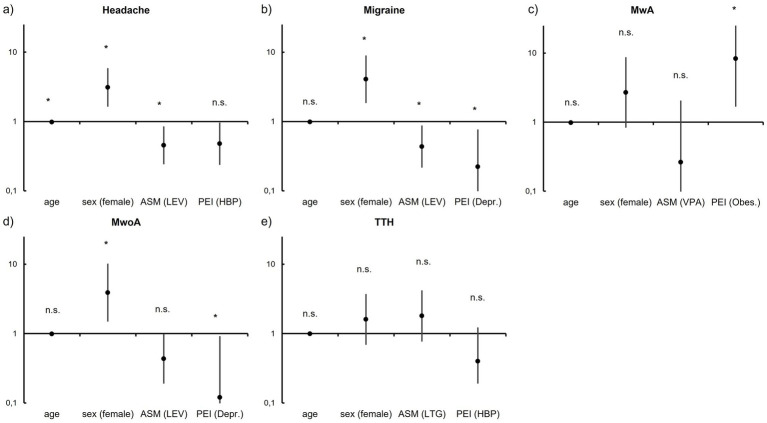
Clinical variables associated with headache and headache subtypes. Odds ratios (OR) of clinical variables associated with headache disorders are given as black dots on vertical lines, representing the 95% confidence interval (CI) of the respective variable. The horizontal line marks an OR value of 1. For groups of variables, the variable with the, respectively, lowest *p*-value is depicted. Tests were calculated for **(a)** headache in general, **(b)** migraine overall, **(c)** migraine with aura (MwA), **(d)** migraine without aura (MwoA), and **(e)** tension-type headache (TTH). Please note that for the variable “age,” OR values are derived from binomial logistic regression. PEI, pre-existing illness; HBP, elevated blood pressure; Depr., depression; Obes., obesity; *, significant; “n.s.”, not significant.

Associations between clinical variables and the presence of any headache disorder were analyzed using Fisher’s exact tests and logistic regression (for age as a continuous variable). Detailed statistical results are provided in the Supplementary material. Odds ratios for the most relevant variables are illustrated in Figure 3, and Table 2 summarizes statistically significant associations for headache and headache subtypes.

**Table 2:** Significant associations with headache and headache subtypes.

Headache disorder	*p*-value	Odds ratio (95%-CI)	Cramér’s *V*
Headache			
Epilepsy	0.016	0.404 (0.199–0.822)	0.198
Focal epilepsy	0.040	0.496 (0.264–0.933)	0.171
Functional/dissociative seizures	0.022	3.310 (1.160–9.450)	0.182
Age^*^	0.028	0.980 (0.963–0.998)	-
Sex (female)	<0.001	3.100 (1.640–5.890)	0.274
Levetiracetam	0.018	0.453 (0.241–0.853)	0.193
Migraine			
Epilepsy	0.007	0.352 (0.171–0.724)	0.226
Focal epilepsy	0.005	0.356 (0.175–0.723)	0.227
Lesional focal epilepsy	0.008	0.314 (0.129–0.763)	0.206
Functional/dissociative seizures	0.019	3.180 (1.270–7.970)	0.199
Sex (female)	<0.001	4.070 (1.850–8.970)	0.283
Levetiracetam	0.023	0.435 (0.216–0.877)	0.184
Valproic acid	0.047	0.332 (0.109–1.010)	0.157
Depression	0.014	0.222 (0.064–0.769)	0.199
Frontal seizure onset^#^	0.024	0.121 (0.016–0.948)	0.219
Migraine with aura			
Epilepsy	0.007	0.215 (0.073–0.630)	0.234
Focal epilepsy	0.002	0.153 (0.042–0.560)	0.247
Lesional focal epilepsy	0.003	0.057 (0.003–0.976)^+^	0.221
Obesity	0.021	8.310 (1.680–41.20)	0.236
Migraine without aura			
Sex (female)	0.004	3.890 (1.490–10.20)	0.228
Depression	0.018	0.120 (0.016–0.916)	0.188

CI, confidence interval.

* Calculated with binomial logistic regression.

# Calculated within the subgroup of epilepsy patients.

+ Calculated using Haldane–Anscombe correction.

In logistic regression analysis, increasing age was significantly negatively associated with headache (*χ*^2^ *p* = 0.028, OR 0.980, Nagelkerke pseudo-*R*^2^ = 0.04), indicating a modest decrease in headache probability with advancing age. Female sex was strongly positively associated with headache (*p* < 0.001, OR 3.10).

Among antiseizure medications, levetiracetam was significantly negatively associated with headache (*p* = 0.018, OR 0.453). Valproic acid showed a non-significant trend toward a negative association (*p* = 0.071, OR 0.459).

Pre-existing illnesses showed no significant associations with headache overall. However, arterial hypertension (*p* = 0.051, OR 0.478) and traumatic brain injury (*p* = 0.114, OR 0.248) demonstrated non-significant trends toward negative associations.

Within the epilepsy subgroup (*n* = 115), neither localization of seizure onset nor seizure type was significantly associated with headache.

### 3.6 Variables associated with migraine, migraine with aura, and migraine without aura

Female sex was significantly positively associated with migraine (*p* < 0.001, OR 4.07) and migraine without aura (*p* = 0.004, OR 3.89). Age showed a non-significant trend toward a negative association with migraine and its subtypes.

Use of levetiracetam (*p* = 0.023, OR 0.435) and valproic acid (*p* = 0.047, OR 0.332) was significantly negatively associated with migraine. Levetiracetam also showed a non-significant trend toward a negative association with migraine without aura (*p* = 0.063, OR 0.433), as did topiramate with migraine overall (*p* = 0.065, OR 0.245).

Among pre-existing illnesses, depression was significantly negatively associated with migraine (*p* = 0.014, OR 0.222) and migraine without aura (*p* = 0.018, OR 0.120). A history of cerebrovascular accident showed a non-significant trend toward a negative association with migraine (*p* = 0.158, OR 0.322). In contrast, obesity was significantly positively associated with migraine with aura (*p* = 0.021, OR 8.31).

Within the epilepsy subgroup, frontal localization of seizure onset was significantly negatively associated with migraine (*p* = 0.024, OR 0.121) and showed a non-significant trend toward a negative association with migraine without aura (*p* = 0.116, OR 0.179). Temporal seizure onset showed non-significant trends toward positive associations with migraine (*p* = 0.359, OR 1.7) and migraine without aura (*p* = 0.208, OR 2.04). No significant associations were observed between seizure types and migraine or its subtypes, and effect sizes were small.

### 3.7 Variables associated with tension-type headache

No clinical variables showed a statistically significant association with tension-type headache (TTH).

Non-significant trends toward positive associations were observed for lamotrigine (*p* = 0.177, OR 1.8) and topiramate (*p* = 0.209, OR 2.2). Arterial hypertension showed a non-significant trend toward a negative association with TTH (*p* = 0.158, OR 0.4), whereas a history of cerebrovascular accident showed a non-significant trend toward a positive association (*p* = 0.174, OR 2.25).

Within the epilepsy subgroup, no significant associations were observed between TTH and seizure type or localization of seizure onset.

## 4 Discussion

We present a dataset of patients with rigorously diagnosed epilepsies and differential conditions, combined with classification-based headache assessment, providing a detailed overview of clinically relevant patient characteristics. We found evidence of a stronger association between headache disorders, particularly migraine, and non-epilepsy patients, especially those with functional/dissociative seizures, than with epilepsy. Notably, only a minority of patients with headache had received a prior diagnosis or treatment in accordance with current clinical guidelines. Younger age and female sex were identified as demographic risk factors for headache, highlighting the importance of screening for comorbidity in these patient populations. ASM were either neutral or negatively associated with headache prevalence, particularly LEV and VPA, suggesting potential protective or therapeutic effects. In contrast, seizure localization and seizure type did not appear to influence headache prevalence.

In this study of patients undergoing comprehensive evaluation in a tertiary epilepsy monitoring unit, headache disorders were highly prevalent, affecting more than half of the cohort. Migraine was the most common headache subtype, followed by tension-type headache. Notably, headache disorders—particularly migraine—were significantly more frequent in patients without epilepsy, especially in those with functional/dissociative seizures, whereas epilepsy was negatively associated with migraine in the overall cohort.

The elevated migraine prevalence in epilepsy patients is consistent with previous studies demonstrating substantial comorbidity between epilepsy and migraine (3, 6, 8). However, in contrast to many reports suggesting a positive association between the two conditions, epilepsy in our cohort was negatively associated with migraine when compared with non-epilepsy patients. This finding is likely influenced by the composition of the comparison group, which consisted of patients with paroxysmal neurological and functional disorders rather than healthy controls. Notably, patients with FDS showed particularly high migraine prevalence, consistent with prior reports and supporting the concept of shared susceptibility related to altered pain processing, stress regulation, and psychiatric comorbidity.

Migraine with aura was not disproportionately represented among epilepsy patients, and its relative frequency was similar to that observed in migraine populations overall (21). This argues against a strong clinical coupling between migraine aura and epileptic seizures in our cohort, despite shared pathophysiological mechanisms such as cortical hyperexcitability and ion channel dysfunction (22–24). Headache disorders were more common in generalized epilepsies than in focal epilepsies, which may reflect genetic susceptibility (15) or demographic factors such as younger age and aligns with previous studies (7, 25).

Tension-type headache was less frequent than migraine, despite its higher prevalence in the general population (26). This discrepancy may reflect underdiagnosis, as tension-type headache is less specific clinically and was rarely diagnosed prior to admission in our cohort. These findings highlight the potential for underrecognition of headache disorders in patients with paroxysmal neurological conditions.

Female sex was a strong predictor of migraine, consistent with established epidemiological patterns (26). Younger age was associated with higher headache prevalence, further supporting known demographic risk factors. Obesity was positively associated with migraine with aura, whereas depression and arterial hypertension showed negative associations with certain headache outcomes. These findings may reflect treatment effects, demographic confounding, or underreporting and should be interpreted cautiously.

Antiseizure medications, particularly levetiracetam and valproic acid, were negatively associated with migraine, supporting their potential protective or therapeutic effects. This aligns with their known or suspected efficacy in migraine prevention and suggests that commonly prescribed antiseizure medications do not worsen headache burden and may confer benefit (27–29). Seizure localization and seizure type showed no consistent associations with headache disorders, indicating that headache comorbidity is unlikely to depend on specific epileptic network characteristics.

Overall, our findings emphasize the importance of headache disorders as a frequent and clinically relevant comorbidity in patients with epilepsy and, in particular, in those with functional/dissociative seizures.

### 4.1 Study limitations

This study has several limitations. First, its retrospective design introduces the possibility of recall and reporting bias, particularly regarding headache history and psychiatric comorbidity. Second, the sample size was limited, reducing statistical power for subgroup analyses and limiting generalizability. Third, the comparison group consisted of patients with other neurological and functional disorders rather than healthy controls, which may have influenced observed associations.

Additionally, the exploratory design and lack of correction for multiple comparisons increase the risk of type I error. Medication exposure was assessed cross-sectionally, precluding conclusions about causal effects. Finally, detailed longitudinal data on headache characteristics and temporal relationships with seizures and treatment were not available.

Prospective studies with larger cohorts and healthy control groups are needed to better characterize headache comorbidity in epilepsy and functional seizures.

## 5 Conclusion

Headache disorders, particularly migraine, are common in patients undergoing evaluation for paroxysmal neurological disorders. Migraine was more prevalent in patients with functional/dissociative seizures than in those with epilepsy, while epilepsy patients still showed higher migraine prevalence than expected in the general population.

Female sex, younger age, and obesity were associated with increased migraine risk, whereas antiseizure medications such as levetiracetam and valproic acid were associated with lower migraine prevalence.

These findings highlight the importance of systematic headache screening, particularly in patients with functional/dissociative seizures and epilepsy. Improved recognition and treatment of headache disorders may help reduce overall disease burden and improve patient outcomes. Future prospective studies should further investigate shared mechanisms and optimize management strategies for patients with comorbid epilepsy, functional seizures, and headache disorders.

## Data Availability

The original contributions presented in the study are included in the article/Supplementary material, further inquiries can be directed to the corresponding author.

## Footnotes

1 Retrieved from https://www.jamovi.org.
